# NCI-60 Whole Exome Sequencing and Pharmacological CellMiner Analyses

**DOI:** 10.1371/journal.pone.0101670

**Published:** 2014-07-17

**Authors:** William C. Reinhold, Sudhir Varma, Fabricio Sousa, Margot Sunshine, Ogan D. Abaan, Sean R. Davis, Spencer W. Reinhold, Kurt W. Kohn, Joel Morris, Paul S. Meltzer, James H. Doroshow, Yves Pommier

**Affiliations:** 1 Developmental Therapeutic Branch, Center for Cancer Research, National Cancer Institute, National Institutes of Health, Bethesda, Maryland, United States of America; 2 HiThru Analytics LLC, Laurel, Maryland, United States of America; 3 Centro de Estudos em Células Tronco, Terapia Celular e Genética Toxicológica, Programa de Pós-Graduação em Farmácia, Universidade Federal de Mato Grosso do Sul, Campo Grande, Mato Grosso do Sul, Brazil; 4 SRA International, Fairfax, Virginia, United States of America; 5 Genetics Branch, Center for Cancer Research, National Cancer Institute, National Institutes of Health, Bethesda, Maryland, United States of America; 6 Developmental Therapeutic Program, Center for Cancer Research, National Cancer Institute, National Institutes of Health, Bethesda, Maryland, United States of America; University of Southern California, United States of America

## Abstract

Exome sequencing provides unprecedented insights into cancer biology and pharmacological response. Here we assess these two parameters for the NCI-60, which is among the richest genomic and pharmacological publicly available cancer cell line databases. Homozygous genetic variants that putatively affect protein function were identified in 1,199 genes (approximately 6% of all genes). Variants that are either enriched or depleted compared to non-cancerous genomes, and thus may be influential in cancer progression and differential drug response were identified for 2,546 genes. Potential gene knockouts are made available. Assessment of cell line response to 19,940 compounds, including 110 FDA-approved drugs, reveals ≈80-fold range in resistance versus sensitivity response across cell lines. 103,422 gene variants were significantly correlated with at least one compound (at p<0.0002). These include genes of known pharmacological importance such as IGF1R, BRAF, RAD52, MTOR, STAT2 and TSC2 as well as a large number of candidate genes such as NOM1, TLL2, and XDH. We introduce two new web-based CellMiner applications that enable exploration of variant-to-compound relationships for a broad range of researchers, especially those without bioinformatics support. The first tool, “Genetic variant versus drug visualization”, provides a visualization of significant correlations between drug activity-gene variant combinations. Examples are given for the known vemurafenib-BRAF, and novel ifosfamide-RAD52 pairings. The second, “Genetic variant summation” allows an assessment of cumulative genetic variations for up to 150 combined genes together; and is designed to identify the variant burden for molecular pathways or functional grouping of genes. An example of its use is provided for the EGFR-ERBB2 pathway gene variant data and the identification of correlated EGFR, ERBB2, MTOR, BRAF, MEK and ERK inhibitors. The new tools are implemented as an updated web-based CellMiner version, for which the present publication serves as a compendium.

## Introduction

Exome sequencing has been recently used for molecular diagnosis and identification of underlying disease gene mutations [1], [2], [3], [4]. In the cancer context, its uses have included identification of low and high-penetrance mutations in cancer-susceptibility genes and mutations associated with clinically relevant phenotypes, such as drug sensitivity [5], [6], [7], [8]. In the context of pharmacology and therapeutics, where both germline and somatic variants are of importance [9], [10], the results of exome sequencing have been proposed for use in precision oncology [11], although there is recognition of the need for increased expertise to achieve clinically actionable information [12].

Cancerous cell lines provide test cases to improve understanding of cancer physiology and pharmacological response, with the potential for rapid translational application. Recent cancer cell studies are providing proof-of-principle by identifying genomic biomarkers to targeted pharmacological agents [11], [13], [14], [15], [16]. The sixty cell lines of the US National Cancer Institute was the first cell line panel set up to explore drug responses, including 9 tissues of origin including refractory tumors such as lung, ovarian, colon, breast, brain and renal cancers, and melanomas together with more treatable cancers such as leukemias [17], [18], [19]. This was done by the Developmental Therapeutics Program (DTP) [20]. Among the NCI-60, two cell line pairs (M14 and MDA-MB-435, and SNB-19 and U251) have subsequently been found to have genotypic similarity [21]. One line, NCI-ADR-RES, is an adriamycin-resistant derivative of the parental OVCAR8 [15]. Over the years, the DTP drug database has grown to >100,000 compounds including an up-to-date list of FDA-approved anticancer drugs as well as several hundred investigational drugs as they emerge from the cancer drug development pipeline [15], [19]. It is by far the largest publicly accessible compound and drug database worldwide [16]. At the same time, the NCI-60 is also the publicly available cancer cell line panel database with the most complete analyses of gene expression [15], [22] and the only cell line panel with publicly available whole exome sequencing (WES) data [11], [21]. Identification of cancer specific variants in the NCI-60 WES provided several pharmacogenomics correlations [11]. These data are freely available in different formats, including BAM [11], CellMiner [23] and Ingenuity [24].

Additional large cell line-drug databases have been developed recently, including the Cancer Cell Line Encyclopedia (CCLE) from the BROAD Institute [25], and the collaborative Wellcome Trust Sanger-Massachusetts General Hospital Genomics of Drug Sensitivity in Cancer (GDS) project [26]. The larger number of cell lines in these datasets provides increased ability to identify rare cancer genomic alterations on a larger number of tissues of origin as well as disease subtypes. Notably, 56 and 44 cell lines are in common with the NCI-60 in the GDS and CCLE, respectively, which enables data cross-validation and expansion studies. In the arena of drugs and compounds, however, the NCI-60 provides data on 19,940 as compared to 24 for CCLE and 138 for CPG. The 19,940 compounds include 110 FDA-approved and 53 clinical trial drugs, as well as 337 with known mechanism of action.

For the purpose of systems molecular biology and pharmacological analyses, accuracy and range of available data are of importance. For the CCLE and GDS, transcript levels are dependent on the results of one platform each, (the Affymetrix U133+2 and HT-HG-U133A v2, respectively). For the NCI-60 there are currently 5 platforms, yielding the opportunity for internal quality and consistency control [15]. Additional forms of information available for the NCI-60 include: i) karyotypic analysis with multiple parameters of genomic instability [27], ii) array comparative genomic hybridization (aCGH) [28], [29], [30], [31], iii) single nucleotide polymorphisms [32], iv) DNA genomic variation [21], v) DNA fingerprinting [33], vi) microRNA expression [22], [34], vii) reverse-phase protein lysate microarrays [35], viii) global proteomic analysis [36], and ix) metabolite profiling [37]. Specialized profiling includes: i) identification of putative tumor stem cell markers [38], ii) HLA class I and II genotyping [39], iii) and quantitative RT-PCR expression of nuclear receptors [40], and ABC transporters [41]. Phenotypic assays include: i) response to ionizing radiation [42], ii) identification of homologous recombination and base excision repair phenotypes [43], iii) CD95 Type I or II status and apoptotic sensitivity [44], iv) DNA damage-induced S-1 phase arrest [45], and v) rhodamine efflux [46]. This is not meant to be an exhaustive list of prior work, but to establish that the NCI-60 has the broadest set of features for systems biology and pharmacology of any data panel.

In the current study, we extend our initial NCI-60 WES analysis [11] with the inclusion of the normal vs. cancer-specific variants, and the introduction of two new genomic and pharmacological tools that complement the existing CellMiner suite. Included are both homozygous variants absent in the normal genomes, and enriched or depleted variants present in the normal genomes. Drug and compound analyses are extended, both for the cell lines independently, and for their integration with the genetic variants. The new CellMiner tools extend the prior basic data accessibility functionalities [23], enabling researchers with limited bioinformatics support to mine the NCI-60 WES data, and facilitate its comparison with other genomic and pharmacological parameters for the NCI-60. The “Genetic variant versus drug visualization” tool allows the user to quickly and easily compare any compound:gene pairing of interest to them, compiling the relevant data into a single output, and assessing their correlation. The “Genetic variant summation” tool extends the prior basic data retrieval functionality by providing a rapid synopsis of all the protein affecting variants from 1–150 genes within any user-defined set. This both provides a snapshot of mutational burden on these genes, and organizes the result into our standard format for easy comparison to all other molecular and pharmacological parameters.

## Materials and Methods

### Cell lines, whole exome sequencing of variants in the NCI-60, and data access

**Figure 1:**
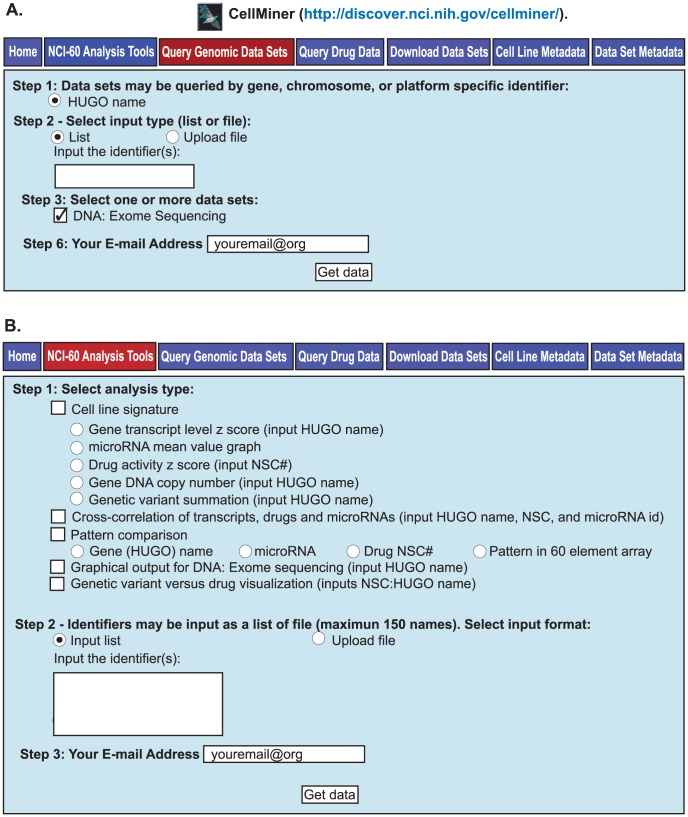
The two tabs for retrieving WES data in CellMiner. **A**. The Query Genomic Data Sets tab. All exome data for a gene may be accessed at http://discover.nci.nih.gov/cellminer/ under the “Query Genomic Data Sets” tab. HUGO name may be selected in Step 1, and List in Step2. The gene identifiers (up to 150 per query) are entered as HUGO names, also in Step 2. The data set, DNA:Exome Sequencing is entered in Step 3. Enter your email address in Step 6, and click “Get data” to receive the output (as an Excel file). **B**. The NCI-60 Analysis Tools tab. Five forms of synopsis data are available for selection in Step 1; Cell line signatures [15], Cross-correlation [15], Pattern comparison [15], Graphical output for DNA:Exome sequencing [15], and Genetic variant versus drug visualization (Figure 5). Identifiers are entered in Step 2. Enter your email address in Step 3, and click “Get data”.

The cell lines used in this study were obtained from the Developmental Therapeutics Program (DTP), and have been described previously [20] [17] [18]. The sequencing technique has been described previously [11]. In brief, 38 Mb of coding region for each cell line was captured using the Agilent SureSelect All Exon v1.0 Kit (Agilent) from sheared DNA libraries were generated. The size-selected samples were sequenced as paired-end 80-mer reads on an Illumina Genome Analyzer IIx instrument (Illumina) following the manufacturer's protocol. Data may be accessed as described in Figure 1.

### Annovar determinations

**Figure 2:**
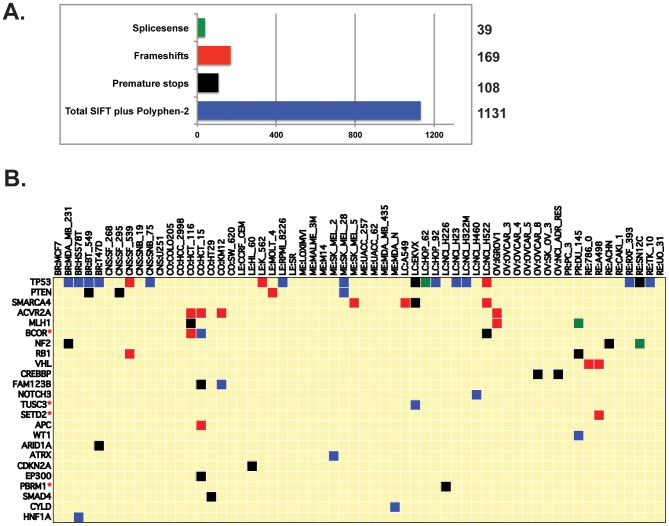
Homozygous, amino-acid changing, putative protein-function-affecting genetic variants present in the NCI-60, and absent in the 1000 Genomes and ESP5400. **A**. The four categories of protein-function-affecting variants, and their level of occurrence. The x-axis is the number of variants in each category, with exact numbers given to the right. **B**. Potential knockout cell lines for tumor suppressors. The x-axis indicates the cell lines. The y-axis indicates the tumor suppressors. Green, red, black, and blue square indicate the presence of homozygous splicesense, frameshift, premature stop, and SIFT or PolyPhen-2 knockouts, respectively (as in A). Additional potential knockouts for the whole genome across the NCI-60 can be readily found in Table S1.

The sequence alignment to the hg19 reference genome, variant call annotation, amino acid number identification and variant effect (used in Figure 2 and subsequently), determination of variant frequency in the 1000 Genomes [47] and ESP5400 [48], scores for Sorting Intolerant From Tolerant (SIFT) [49], and Polyphen-2 [50], and presence or absence in COSMIC [51] were done within Annovar [52], and have been described previously [11].

### Variant identifiers, reference sequence accession numbers, and frequency of variants

Previously identified variants are defined by the National Center for Biotechnology Information (NCBI) database of Single Nucleotide Polymorphisms site (dbSNP) [53]. The accession number is for the reference sequence from the NCBI used in determining amino acid change affects [54]. For comparison to the NCI-60 variants, we used the ESP5400 (downloaded December 20, 2011).

**Figure 3:**
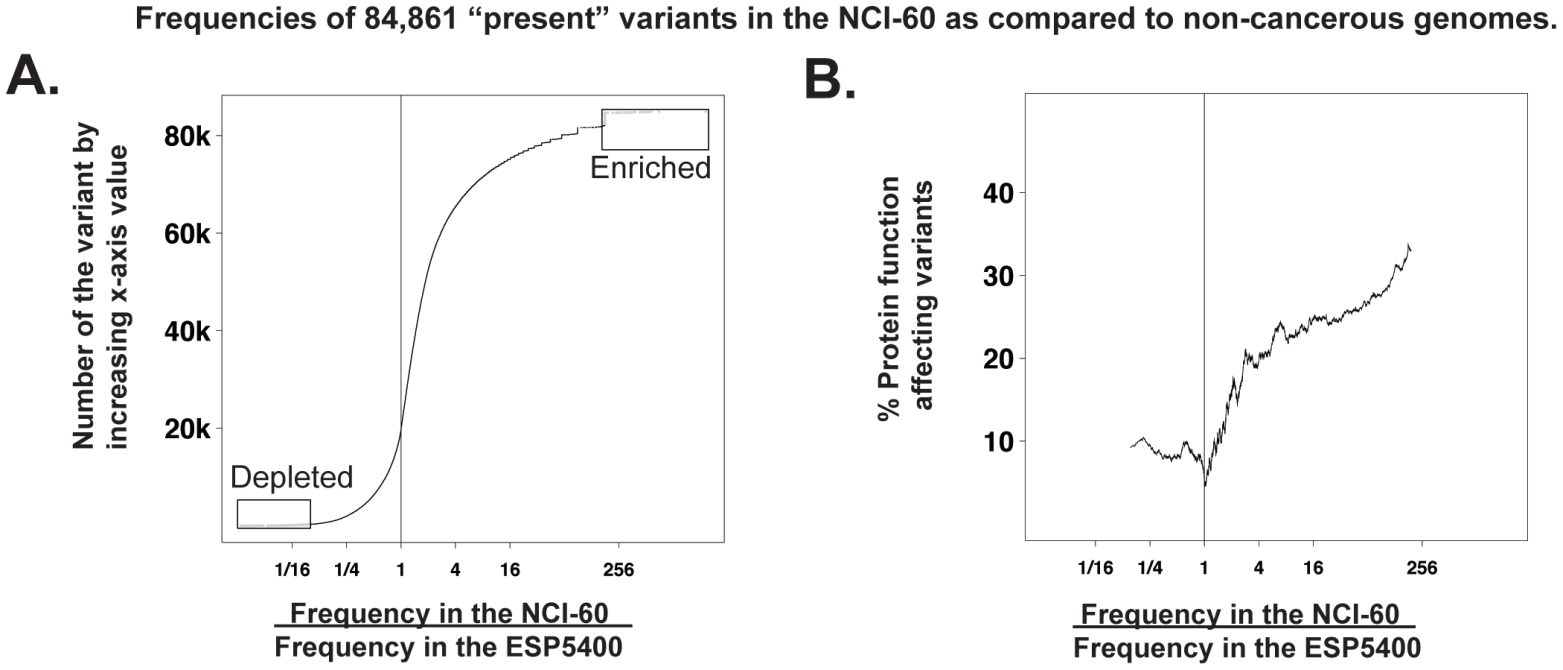
Comparisons of variant frequencies in the NCI-60 to that in non-cancerous tissues (the ESP5400). **A**. Scatter plot for all 84,861 variants that occur both in the NCI-60 and the ESP5400. The x-axis is the ratio of frequencies of variants in the NCI-60 vs. the frequencies of the same variants in the ESP5400. The y-axis is the number of the variants, ordered by the frequency ratio. The boxed “Enriched” variants (in the NCI-60) include 2,792 variants, and the boxed “Depleted” variants numbers 319. Enrichment is defined as the top 2.5% of variants for which the ratio of frequencies is ≥10. Depletion is defined as the bottom 2.5% of variants, for which the ratio of frequencies is ≤0.1. In both A and B, the vertical lines drawn at x = 1 indicate equal frequencies in the NCI-60 and non-cancerous genomes (ESP5400). **B**. Scatter plot for the protein-function-affecting variants that occur in both the NCI-60 and the non-cancerous genomes. The y-axis is the percent of protein function affecting amino-acid changing variants (as compared to all variants) within a sliding window of size 2001.

The frequency of variants present in the ESP5400 in Figure 3A and B is calculated as an average of the allelic frequencies for each cell line (as determined by Annovar). The number of genomes in the ESP5400 was 5400. The 1000 Genomes were not included in this calculation due to their being a different and variable form of data (whole genome sequencing) as opposed to exome sequencing (as for the NCI-60 and ESP5400) that might distort the ratio of frequencies in Figure 3A and B.

### Determination of statistical enrichment, overrepresentation, and underrepresentation

Calculation of enrichment for cancer-driver genes in Figure 3 and Table S2 was done using Fishers exact testing. For enriched and depleted groups of “present” variants in Figure 3A, the 2.5% cutoff occurred within a large group with equivalent ratios, all of which were included. The determination of the percent of protein function affecting variants in Figure 3B was done using a sliding window of 2001 (variants).

### Quantitation of drug activities

The 50% growth inhibitory levels (GI50s) at 48 hours were determined by the Developmental Therapeutics Program (DTP) [20] as described previously [55]. All GI50 data was assessed and transformed to z scores as described previously [15], yielding patterns for 19,940 compounds (CellMiner version 1.4 [23]).

### Comparison of variants and drug activities

The combination of up to five variants with the highest correlation to each compounds activity was identified. Only variants that were “amino-acid changing” were included; defined as missense, splice-sense, frameshift, read-through, non-frameshift insertion or deletion (nucleotides are changed in factors of 3), or premature stops. Other criteria for inclusion of variants for comparison to drug activity are, i) there are <13 cell lines with any single variant, ii) the combination identified for a gene were associated to the response of at least three cell lines, iii) they did not occur in segmental duplications (assessed using Annovar), and iv) they did not map to more than one genomic location (using BLAT [56]). Drugs activities were considered shifted for a cell line if they were changed from the 60-cell line mean by 0.5 standard deviations.

The Matthews correlation coefficient (MCC) was calculated as follows: 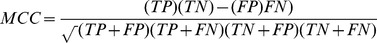



*TP* is the number of true positives (number of cell lines that have the variant and respond to the drug), *FP* number of false positives (number of cell lines that have the variant but do not respond to the drug), *FN* the number of false negatives (number of cell lines that do not have the variant but respond to the drug), and *TN* the number of true negatives (number of cell lines that do not have the variant and do not respond to the drug).

Precision is the fraction of the variant(s)-containing cell lines that also have the drug response, calculated as: TP/(TP+FP).

Recall is the fraction of cell lines with drug response that also have variant(s), calculated as FN/(TP+FP).

### The “Genetic variant summation” web-based tool

The summation of variants for an individual gene is derived from the observed level of the variant. If there are multiple variants present, these probabilities are considered to be independent. The maximum effect of a single gene's variants may go to 100% for any cell line, and are calculated as follows. 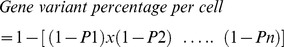



**P1** =  Percentage for variant 1

**P2** =  Percentage for variant 2

**Pn** =  Percentage for variant n

The summation of amino acid changing variants for multiple genes is the sum of the individual genes values for each cell line (with no maximum value).

### Composite correlations for gene's variants versus compounds based on presence or absence in the non-cancerous genomes

For the comparison of variants either present or absent in the non-cancerous genomes to drug response *in toto*, the gene's variants (from one to five) that yielded the highest significant correlations for each of the compounds were characterized as being either present or absent in the non-cancerous genomes, totaled, and their proportions determined at four p value thresholds (p<0.05, 2×10^−4^, 1×10^−6^, and 1×10^−8^ using Matthew's correlation).

## Results

### Variants in the NCI-60 and new CellMiner tool to facilitate their retrieval and interpretation

Of the 140,171 individual genetic variants identified [11], 86,887 (62%) are “present” in either the 1000 Genome project or the ESP5400 non-cancerous genomes, whereas 53,284 (37%) are absent (cancer cell associated). As described previously [11], the data are accessible through our CellMiner web-based tool [23] with tabular data for individual genes retrieved at the “Query Genomic Data” tab by checking “DNA:Exome Seq” in Step 3 [see Figure 1A [11]]. Graphical data can be retrieved at the “NCI-60 Analysis Tools” tab using the “Graphical output for DNA: Exome sequencing” tool [see Figure 1B [11]].

### Catalog of homozygous deleterious variants across the NCI-60

Among the variants, we catalogued a subset of 1,447 that are: i) homozygous, ii) absent from the 1000 Genomes and the ESP5400, iii) alter the amino acid sequence and iv) likely to affect protein function. The criteria for affecting protein function were the introduction of i) splicesense changes, ii) frameshifts, iii) premature stops, or iv) missense changes with SIFT values <0.05, or Polyphen-2 (values ≥0.85). The numbers of variants that fit each of these criteria are presented in Figure 2A.

In all, 1,199 genes contain variants from at least one of the four categories. A subset of putative function affecting mutations that occur in cancer-relevant genes is presented in Table 1, with the complete listing of 1,447 variants (that fit the above criteria) in Table S1. Tumor suppressor homozygous variants are presented in Figure 2B, including several not previously described (BCOR, SETD2, TUSC3 and PBRM1) in either the Cancer Cell Line Encyclopedia [13] or the Sanger Cancer Genome Project [14] [57]. The 1,199 gene set (Table S1) are significantly enriched (p<2×10^−24^) for previously recognized cancer-driver genes [58].

**Table 1:** Homozygous variants that putatively affect protein function and are absent in the 1000 Genomes and the ESP5400^a^.

Gene name^b^	Change type	AA impact^c^	Unique identifier^d^	SNP id^e^	SIFT score^f^	Polyphen^g^	Accession #^h^	Cell lines in which variants occur
TP53	SpliceSence	-	chr17:7577610_T_C	-	NA	NA	NM_001126114	LC:HOP_62
MLH1	SpliceSence	-	chr3:37038108_A_T	-	NA	NA	NM_000249	PR:DU_145
FANCM	SpliceSence	-	chr14:45628296_*_-TA	-	NA	NA	null	ME:LOXIMVI
TRAF1	SpliceSence	-	chr9:123667518_T_G	-	NA	NA	NM_005658	LC:EKVX
FLT3	SpliceSence	-	chr13:28622411_C_A	-	NA	NA	NM_004119	LC:NCI_H322M
RB1	Frameshift	116_117del	chr13:48916816_*_-ACTT	-	NA	NA	NM_000321	CNS:SF_539
APC	Frameshift	G1398fs	chr5:112175539_*_-C	-	NA	NA	NM_001127511	CO:HCT_15
PTEN	Frameshift	K267fs	chr10:89717775_*_-A	rs121913289	NA	NA	NM_000314	LE:MOLT_4
VHL	Frameshift	G104fs	chr3:10183842_*_-G	-	NA	NA	NM_000551	RE:786_0
CASP10	Frameshift	334_336del	chr2:202074072_*_-GCCAAGG	-	NA	NA	NM_001206524	BR:BT_549
SMAD4	Premature Stop	Q311X	chr18:48586262_C_T	-	0.35	0.735	NM_005359	CO:HT29
CDKN2A	Premature Stop	R80X	chr9:21971120_G_A	rs121913388	0.84	0.737	NM_000077	LE:HL_60
MAP3K5	Premature Stop	S1240X	chr6:136888811_G_C	-	0.04	0.735	NM_005923	LC:NCI_H322M
CDH4	Premature Stop	Q383X	chr20:60485547_C_T	-	0.13	0.735	NM_001252338	LC:NCI_H322M
NF2	Premature Stop	R57X	chr22:30032794_C_T	rs121434259	1	0.735	NM_000268	RE:ACHN
ABCB1	SIFT and PolyPhen	A1217T	chr7:87133753_C_T	-	0.00	0.968	NM_000927	ME:SK_MEL_28
ABL1	SIFT and PolyPhen	K7R	chr9:133589726_A_G	-	0.00	0.956	NM_007313	BR:HS578T
AXL	SIFT and PolyPhen	H292Q	chr19:41743941_T_A	-	0.05	0.992	NM_001699	OV:OVCAR_8;OV:NCI_ADR_RES
EGFR	SIFT and PolyPhen	P753S	chr7:55242487_C_T	rs121913231	0.00	0.996	NM_005228	ME:SK_MEL_28
KRAS	SIFT and PolyPhen	G12V	chr12:25398284_C_A	rs121913529	0.01	0.91	NM_004985	CO:SW_620;OV:OVCAR_5
NRAS	SIFT and PolyPhen	Q61L	chr1:115256529_T_A	rs11554290	0.00	0.868	NM_002524	LE:HL_60
PIK3R2	SIFT and PolyPhen	N561D	chr19:18278061_A_G	-	0.02	0.991	NM_005027	ME:LOXIMVI
TDP1	SIFT and PolyPhen	K292E	chr14:90446966_A_G	-	0.00	0.999	NM_001008744	LC:NCI_H522
BRAF	SIFT	V600E	chr7:140453136_A_T	rs113488022	0.00	0.796	NM_004333	ME:SK_MEL_28;ME:UACC_62
WT1	SIFT	S204R	chr11:32456280_G_T	-	0.00	0.023	NM_000378	PR:DU_145

a Variants called as homozygous by Annovar (http://www.openbioinformatics.org/annovar/). Variants absent in both the 1000 Genomes catalog of human genetic variation (http://www.1000genomes.org/) and the ESP5400 (http://evs.gs.washington.edu/EVS/).

b Official gene names as defined by the HUGO Gene Nomenclature Committee (http://www.genenames.org/).

c Amino acid impact. 116_117del indicates a deletion affecting amino acids 116 and 117. G13981fs indicates a change from an amino acid to a frameshift. R80X indicates a change from an amino acid to a stop. K7RD indicates a change in amino acids.

d Denotes the chromosome number, start location, and the nucleotide change. _* indicates that there has been either an addition (+) or a deletion (−) of nucleotides.

e Single nucleotide polymorphisms identifiers as defined at dbSNP (http://www.ncbi.nlm.nih.gov/projects/SNP/).

f Percent presense of the variant in the 1000 Genomes catalog of human genetic variation (http://www.1000genomes.org/).

f Sorting Intolerant From Tolerent (http://sift.bii.a-star.edu.sg/index.html).

g Prediction of functional effects of human nsSNPs, or PolyPhen-2 (http://genetics.bwh.harvard.edu/pph2/).

h Reference sequence accession number from the National Center for Biotechnology Information, NCBI (http://www.ncbi.nlm.nih.gov/).

### Identification of normal variants either enriched or depleted in the NCI-60

There are 84,861 “normal” variants (SNPs) in the NCI-60 that are also present in the ESP5400 database. Their distribution, expressed as their frequencies in the NCI-60 variants/ESP5400 is visualized in Figure 3A. Variants that: i) occur in the top or bottom 2.5% of ratio values, and with ratios greater than 10∶1 (enriched), or smaller than 1∶10 (depleted) for the ESP5400 dataset were identified. This yielded 2,792 enriched and 319 depleted variants. Of these, 1,547 (52.9%) of the enriched, and 88 (73.9%) of the depleted variants affect amino acids in 1,214 and 84 unique genes, respectively. Variants that do not affect amino acid sequence have been included as these changes can still potentially have biological effects.

Figure 3B depicts the frequency of the normal variants that are predicted to affect protein function (as defined in Figure 2A). As variant frequency in the NCI-60 (the x-axis values) increases from a ratio of 1, so does the proportion of those functionally relevant variants (the y-axis values). A subset of the enriched or depleted variants that occur in previously recognized cancer-driver genes [58] is listed in Table 2. The total list of enriched or depleted variants is in Table S2.

**Table 2:** Genes that are either enriched or depleted in the NCI-60), and are cancer-driver genes^a^.

			Variant identification and effect		
Gene name^b^	Functional category^c^	AA impact^d^	Unique identifier^d^	dbSNP ID^e^	NCI-60/ESP5400 frequency^f^
TP53	Tumor suppressor, DDR, Apoptosis	R116Q	chr17:7577538_C_T	rs11540652	538
PIK3CA	Oncogene, Protein kinase		chr3:178928178_C_T		472
TET2	Tumor suppressor	S460F	chr4:106156478_C_T		358
NCOR1		R121C	chr17:16075191_G_A	rs140151735	358
GATA2	Oncogene	V211V	chr3:128204808_G_C		358
SETBP1		V81V	chr18:42281554_G_A		179
ASXL1	Tumor suppressor	A611T	chr20:31022346_G_A		179
APC	Tumor suppressor, Apoptosis	S140G	chr5:112103053_A_G	rs150973053	179
ARID1A	Tumor suppressor	R1053H	chr1:27094450_G_A		179
ARID1B		A1922A	chr6:157528080_G_T		179
PRDM1	Tumor suppressor	A385V	chr6:106553591_C_T		179
ACVR1B		D502D	chr12:52387882_C_T		179
ASXL1	Tumor suppressor	T557T	chr20:31021672_C_T		179
SMARCB1	Tumor suppressor	E201D	chr22:24158958_G_T	rs142218902	179
BAP1	Tumor suppressor		chr3:52439102_C_A	rs186001194	179
BRCA2	Tumor suppressor, DDR, Apoptosis	R2784Q	chr13:32944558_G_A	rs80359076	179
MSH2	Tumor suppressor, DDR	E198E	chr2:47637460_A_G		179
CDH1	Tumor suppressor	V391V	chr16:68847251_C_T	rs148080550	179
RUNX1	Tumor suppressor	V137V	chr21:36252870_G_A		179
MLH1	Tumor suppressor, DDR	E219A	chr3:37067468_A_C		179
TSC1	Tumor suppressor, Protein kinase, Apoptosis		chr9:135782112_C_T		179
BRCA2	Tumor suppressor, DDR, Apoptosis	R2896C	chr13:32950860_C_T		179
NCOR1		S1867S	chr17:15961885_G_A		179
ARID1B		D1713D	chr6:157527453_C_T	rs189662115	179
NF1	Tumor suppressor	L1954L	chr17:29661968_T_C		179
TP53	Tumor suppressor, DDR, Apoptosis	G113S	chr17:7577548_C_T	rs28934575	179
DNMT1		S809S	chr19:10260240_C_T		179
ABL1	Oncogene, Protein kinase, Apoptosis	P986P	chr9:133760635_A_G	rs35445683	179
ATM	Tumor suppressor, DDR, Apoptosis	R32C	chr11:108098524_C_T	rs148061139	179
PTCH1	Tumor suppressor		chr9:98241268_G_A		179
NOTCH2	Tumor suppressor, Apoptosis	H1160R	chr1:120479948_T_C	rs142876168	179
SETBP1		Q1244R	chr18:42533036_A_G		179
SETBP1		P256P	chr18:42530073_C_T	rs141858699	179
NF1	Tumor suppressor	A2596V	chr17:29684089_C_T		179
APC	Tumor suppressor, Apoptosis		chr5:112154629_C_T		179
CDC73	Tumor suppressor		chr1:193091510_G_A		179
MSH2	Tumor suppressor, DDR	D487E	chr2:47690244_C_G	rs35107951	179
ARID1A	Tumor suppressor	A1136A	chr1:27098992_G_A	rs146598030	179
ATM	Tumor suppressor, DDR, Apoptosis	R337C	chr11:108117798_C_T	rs138398778	179
					
ALK	Oncogene	L9L	chr2:30143499_G_C	rs4358080	0.092
KDM6A	Tumor suppressor		chrX:44913052_T_A	rs5952285	0.066
SMARCA4	Tumor suppressor	D1599D	chr19:11170839_T_C	rs7275	0.063

a Genes selected from the boxed fractions from Figure 3A. Cancer-driver genes are as described previosly [58].

b Official gene names as defined by the HUGO Gene Nomenclature Committee (http://www.genenames.org/).

c DDR is DNA damage response, and SLC is solute carrier.

d Amino acid impact. Example, R116Q indicates a change from R (Arg, arginine) to Q (Gln, glutamine).

d Denotes the chromosome number, start location, and the nucleotide change. _* indicates that either an addition (+) or a deletion (−) of nucleotides.

e Single nucleotide polymorphisms identifiers as defined at dbSNP (http://www.ncbi.nlm.nih.gov/projects/SNP/).

f Frequency calculated as described in Materials and Methods.

### Overall drug activity database for the NCI-60

**Figure 4:**
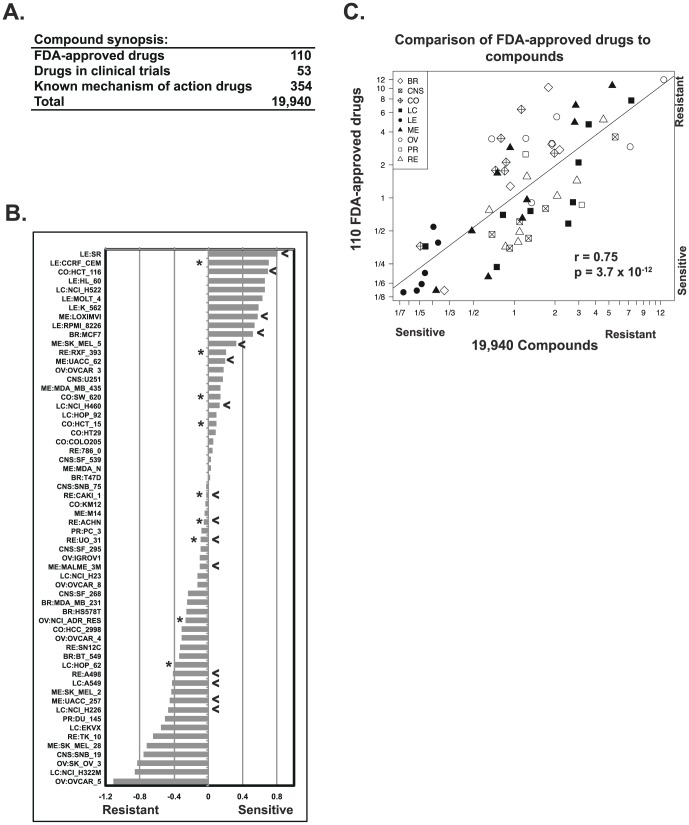
Overall drug responses in the NCI-60. **A**. Compounds and drugs used in the present analyses. **B**. The cellular responses to the 19,940 compounds were categorized for each cell line, as resistant (z score ≤−0.5), no response (z score >−0.5 to <0.5), or sensitive (z score ≥0.5). The number of compounds categorized as leading to sensitivity or resistance was determined for each cell line. The ratio of these resistance:sensitivity determinations (plotted as −log10 values) is on the x-axis. The cell lines are on the y-axis. Asterisks denote ABCB1-positive cells as measured by rhodamine efflux [46]. Arrowheads denote cell lines that are TP53 wild-type. **C**. Scatter plot of resistance:sensitivity ratios for the 19,940 compounds (x-axis) versus the 110 FDA-approved drugs (y-axis). The same ratios of resistance:sensitivity from B were determined for the subset of 110 FDA-approved drugs. Each point is a cell line (plotted as -log values). Tissues of origin are indicated: BR is breast, CNS is central nervous system, CO is colon, LC is lung cancer, LE is leukemia, ME is melanoma, OV is ovarian, PR is prostate, and RE is renal.

Growth inhibition 50% data (GI50, from the DTP) were filtered and converted to z scores [15] for 19,940 compounds (Table S3), including 110 FDA-approved drugs (Figure 4A). Cell lines were considered to be either sensitive or resistant to a compound if their response was shifted by ≥0.5 or ≤−0.5 standard deviations from the 60 cell line mean, respectively. The number of compounds to which each cell line was found to be sensitive or resistant was totaled. Ratios calculated from these two totals (Figure 4B) revealed a 83-fold range between the most sensitive (leukemia SR) and most resistant (ovarian OVCAR-5) cells. Eliminating the leukemias, which are generally most sensitive to drugs, the resistance/sensitivity ratio range remained at 65-fold.

The same analysis for the 110 FDA-approved drugs included in the database yielded a 87-fold range (with or without leukemias). Comparison of all compounds to FDA-approved drugs (Figure 4C) showed a robust statistical significance (r = 0.75, p = 3.7×10^−12^ by Pearson's correlation), indicating cellular response to all compounds is quite similar to the FDA-approved subset. Notably, we did not find significant correlation (r = −0.12) between the drug response ratios and ABCB1 gene expression, or TP53 wild-type versus mutant status (asterisks and arrows, respectively, in Figure 4B) [46]. In addition, we looked for associations to the protein function affecting variants (as defined in Figure 2) for: i) DNA damage response, ii) oncogenes, iii) tumor suppressors, iv) ABC transporters, v) solute carriers, vi) apoptosis genes, and vii) the total number of genes (with protein function affecting mutations) per cell line, but found no significant relationship. However, drug sensitivity was found significantly correlated with cell line doubling-times (p<0.0015) [59], and epithelial (BR, CO, LC, OV, PR, and RE) versus non-epithelial (CNS, LE, and ME) tissue of origin status (p<0.02).

### Comparison of single gene variants with drug responses

The 42,990 amino acid changing variants were compared to the activities of the 19,940 compounds from Figure 4A (∼16.5×10^−9^ compound-gene-cell line combinations). For each gene, the combinatorial of one to five variants that yielded the highest correlation to each compound activity was determined, and Mathew's correlation coefficients (MCC) were computed. Our selection criteria for variants associated with drug response included i) MCC of ≥0.596 (p≤0.0002 for n = 35), ii) precision ≥0.70, iii) overall precision for a gene's variants of ≥0.50, and iv) that three or more cell lines had both the identified variant(s) and a consistent shift in drug response (either more sensitive or resistant; see Materials and Methods for details). Variants were included regardless of presence or absence in the normal genomes, as our goal was to recognize both disease-associated and germline influences on pharmacological response.

Using these criteria, we identified 80,265 increased-sensitivity, and 21,436 increased-resistance gene-drug pairs (Table S4A and B, respectively). For the 19,940 compounds assessed, this includes at least one gene match for 13,891 of the compounds, involving 7,288 unique genes. For the 163 drugs that are either FDA-approved or under clinical trial, we identified 406 increased-sensitivity and 205 increased-resistance gene drug pairs. There are 75 and 58 recognized cancer-driver genes in the increased sensitivity, and resistance listings (Table S4A and B), respectively. Some genes have variations that are associated with multiple compounds, a potential flag for pharmacological importance. These include i) the DNA damage responder RAD52 with 45 sensitive and 0 resistance-correlated compounds, ii) the kinase BRAF with 56 sensitive and three resistance-correlated compounds, iii) the zinc finger domain containing gene ZC3H4 with 229 sensitive and 3 resistant-correlated compounds, and iv) the helicase CHD4 with 976 sensitive and 27 resistance-correlated compounds. A subset of the gene-drug pairs including FDA-approved or clinical trial drugs, and genes of interest in either the cancer or pharmacological context, are shown in Table 1. This is an unbiased mathematical comparison designed to identify relationships between genetic variants and drugs, and the resultant pairings should be considered in the context of biological and pharmacological knowledge. Significant correlation is not a proof of a causal relationship but could be a valuable tool to generate hypotheses.

### Comparison of variants either present or absent in the non-cancerous genomes to drug response *in toto*

To obtain an indication of relative contribution to pharmacological response for the genetic variants, we summed those variants from Table S4A and B (column D, AA Impact) with respect to their presence or absence in the non-cancerous genomes. Based on these summations, the proportion of variants correlated to pharmacological response was 56.7% absent and 43.3%, present (in the normal genomes). This is evidence that more than half of the effect of genetic variants on pharmacological response come from the group absent from the non-cancerous genomes. This occurs despite the absent variants occurring in fewer cell lines, and thus being numerically much less abundant (7.0%) than the present (93.0%). Biologically, this implies that the present variants individually tend to be less influential in their effect on pharmacology; however, this is compensated for due to their numerical predominance.

### A Web-based tool to explore and visualize variant-drug relationships

**Figure 5:**
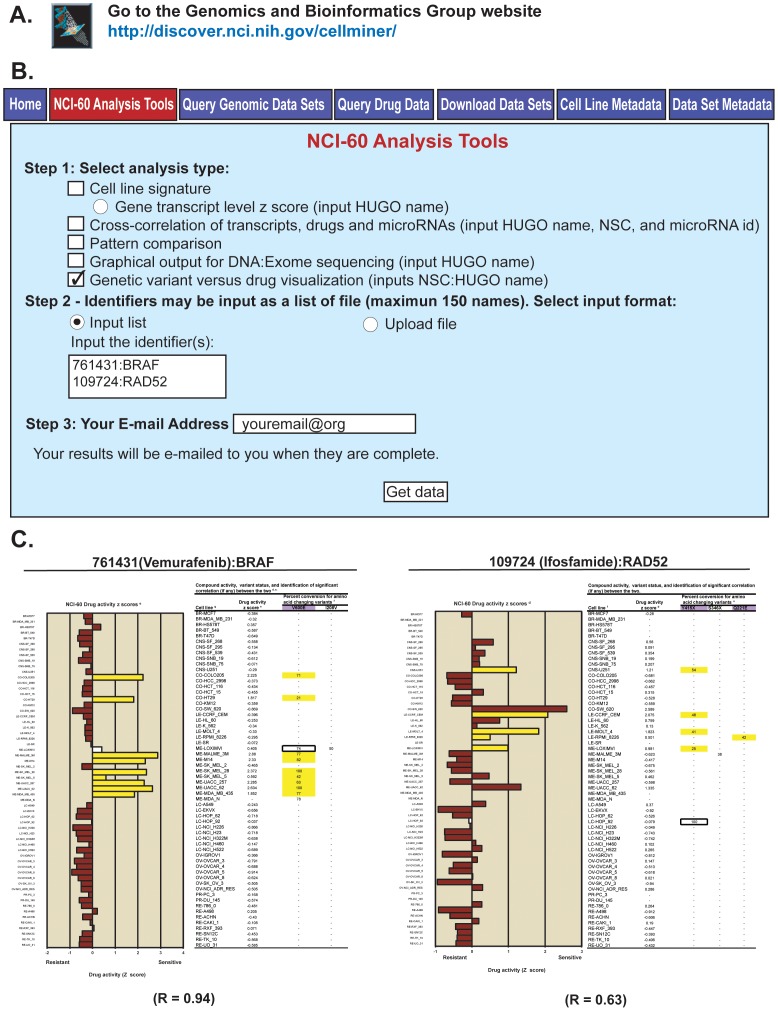
The “Genetic variant versus drug visualization” web-based tool and output examples. **A**. The tool is accessed through our CellMiner web-application at http://discover.nci.nih.gov/cellminer/. **B**. Within the “NCI-60 Analysis Tools” tab (shown in red), the tool is selected by checking the box in Step 1. The compound and gene identifiers (up to 150 pairs) are entered in Step 2, using NSC numbers for the compounds, and HUGO names for the genes. Enter your email address and click “Get data” in Step 3 to receive the output (as an Excel file). **C**. The output incudes a bar-plot of the compound activity z scores. The x-axis is the activity z scores, and the y-axis the NCI-60 cell lines ordered by tissue of origin. The tabular output includes the cell lines (in column 1), the compound z scores (in column 2), followed by the amino acid changing variants. Cell lines whose activities or variant status contribute to a statistically significant relationship are indicated by yellow coloring. For the bar plot, brown fills indicate cell lines for which no variant correlates with a shift in drug activity, and the white fill that the cell line has a variant correlated to a shift in the drug activity, but that that cell does not contribute to the correlation. For the tabular data, the purple filled in headers indicate the variant(s) that have significant correlation to the compound activity. The white box indicates that the cell line contains a variant that correlates to the compound, but that that cell line has no significant shift in drug activity (that is it is less than plus or minus 0.5 standard deviations from the mean at 0).

To facilitate the exploration of potential relationships between gene's variants and compound activities, we created the “Genetic variant versus drug visualization” tool (Figure 5). This tool is accessible through the URL in Figure 5A under the “NCI-60 Analysis Tools” tab, as shown in Figure 5B. The tool is selected in Step1 and the “compound:gene” combinations of interest entered in Step 2 with the compound as NSC number followed by a colon and the HUGO gene name (Fig. 5B); note that up to 150 combinations can be entered in the same query (see example in Fig. 5B). In Step 3, users enter their email address and click “Get data” to receive the output.

For each query, the output, sent by return email within a few minutes, as an Excel file, contains a bar plot of the compound activity z scores (Figure 5C). In addition, there is side-by-side tabular data for these activity values, plus the amino acid changing variant percentages for the input gene for each cell line. Significant correlations (p<0.05) are indicated by yellow coloring of the bars for compound activity, and yellow highlighting of the percent conversions for the variants (Figure 5C). Additional information not shown in Figure 5C include: i) the drug name, ii) the drug mechanism of action (if known), iii) the compound PubChem identifier, iv) the variant genomic location, v) the variants nucleotide and amino acid changes, vi) the SIFT and Polyphen-2 scores for each variant, and whether these are considered to affect protein function, and vii) whether individual variants occur (and at which frequency) in normal genomes (either in the 1000 Genomes or the ESP5400). The two examples in Figure 5 illustrate the validity and usefulness of the approach. As expected, the activity of the BRAF inhibitor, vemurafenib is highly selectively for the BRAF-V600E mutated cell lines, which include most of the melanoma and 2 of the colon cell lines. Other associations can be explored at will. Notably, ifosfamide was correlated with RAD52 mutations (Fig. 5C, right panels).

### A Web-based tool to identify variants for a single gene or in a pathway or functional group

**Figure 6:**
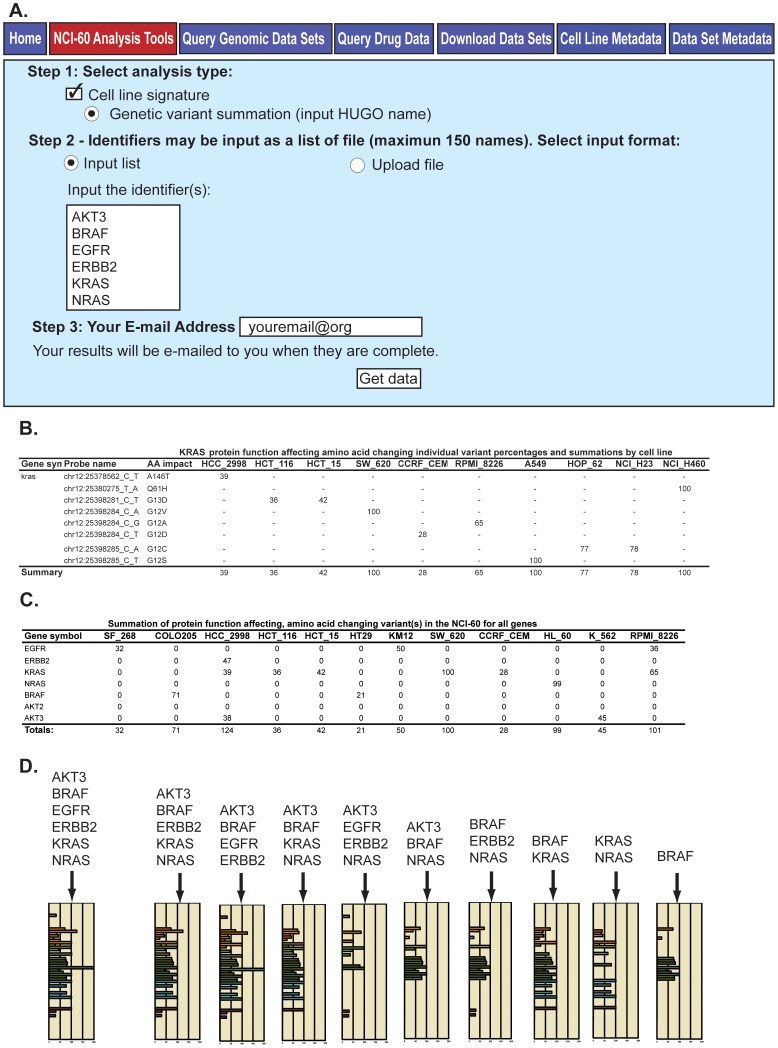
The “Genetic variant summation” tool, and output. **A**. The tool is accessed through CellMiner at http://discover.nci.nih.gov/cellminer/, under the “NCI-60 Analysis Tools” tab as described in Figure 5A. The tool is selected in Step 1, and the gene identifiers (up to 150) are entered as HUGO names in Step 2. Enter your email address and click “Get data” in Step 3 to receive the output (as an Excel file). **B**. The output incudes two versions of the data. The first contains the amino acid changing variants for each input gene. The second contains the subset of these that are included in one of the protein function affecting categories (as defined in Figure 2), and are absent from the non-cancerous 1000 Genomes and ESP5400. Both provide i) chromosome number, ii) nucleotide location and change, iii) amino acid number and change, iv) percent conversion of each cell line for that variant for the NCI-60, and v) the summation of the gene's variants present for each cell line (to a maximum of 100%). The example of KRAS is shown for a subset (due to space constraints) of the cells. **C**. The tool provides a summation of the variants for all genes in the input. The summary values from B for each gene are added together (with no maximum) to provide a measurement of variant burden (see “Totals”, bottom row). **D**. The totals from C are used to create a bar graph. The x-axis is the summation of variants values (“Totals” from C). The y-axis is the cell lines, color-coded by tissue of origin [15], [70]. Several outputs are included for illustration, with the first being from the 6-gene input in A.

As different variants can affect the function of a given gene (for instance TP53 or most tumor suppressor genes), and molecular pathways can be affected by genetic variants present in the different genes constituting the pathway, we designed a tool to both identify and sum those variants. This is the “Genetic variant summation” tool, accessible using the same URL as in Figure 5A, and found under the “NCI-60 Analysis Tools” tab. The tool is used by checking “Cell line signature” and “Genetic variant summation” in Step 1 (Figure 6A). Up to 150 genes (or a single gene) can be queried in a single search either by entering the list of genes (using official HUGO names), or by uploading the genes from a .txt or .xls file in Step 2. In Step 3, users enter their email address, and click “Get data” to obtain the results, which are sent by email within minutes as Excel files.

In the example in Figure 6A, six genes from the pharmacologically important EGFR-ERBB2-RAS pathway were included as input (the tool works similarly well if users only wish to retrieve data for a single gene). For each gene or gene list, the “Genetic variant summation” tool returns an Excel file that lists: i) a complete list of all the variants that affect amino acids (in a first worksheet, “AA Change”), and ii) in a second worksheet (“Prot. Func. Affecting AA Change”), the subset of those variants that are both predicted to affect protein function (based on the criteria listed in Figure 2A) and absent in the non-cancerous 1000 Genomes and the ESP5400. To simplify the presentation, Figure 6B only shows data from this second group (“Prot. Func. Affecting AA Change”) for KRAS and selected cell lines. For each cell line and variant, the numbers indicate the percent conversion for that variant. For instance, in Figure 6B, four of the colon carcinoma cell lines show protein-affecting variants. The classical activating mutations G12V and G13D are observed in 3 cell lines: HCT116, HCT15 and SW620. SW620 score 100 for the G12V mutation indicating homozygous mutation, whereas HCT116 and HCT15 score 36 and 42 for the G13D mutation indicative of heterozygous mutation [11].

In the summary row (illustrated in Fig. 6B), for any given gene, multiple variants can be observed in the same cell line; in which case, the gene variants summation “Summary” is set to a maximum of 100 percent for each cell line to reflect homozygosity (see Materials and Methods).

The Genetic variant summation tool also provides the summation pattern for the whole NCI-60 for the set of genes included in the search (Figure 6D). Several summation pattern examples are given in Figure 6D, for different subsets of gene combinations (see Fig. 6A and Fig. 6D left panel for the 6 gene result, and see right panel in Fig. 6D for BRAF alone). Additional information provided for the variants in the Excel spreadsheet (not shown in Fig. 6) includes: i) SIFT scores, ii) Polyphen-2 scores, iii) type of amino acid affecting change (missense, nonsense, etc.), and iv) percent presence in the 1000 Genomes and ESP5400. Users are invited to explore on their own using our CellMiner tool to fully appreciate the extent of the data made readily available in the Excel result files.

### Use of genetic variation patterns in the exploration of drug response

**Figure 7:**
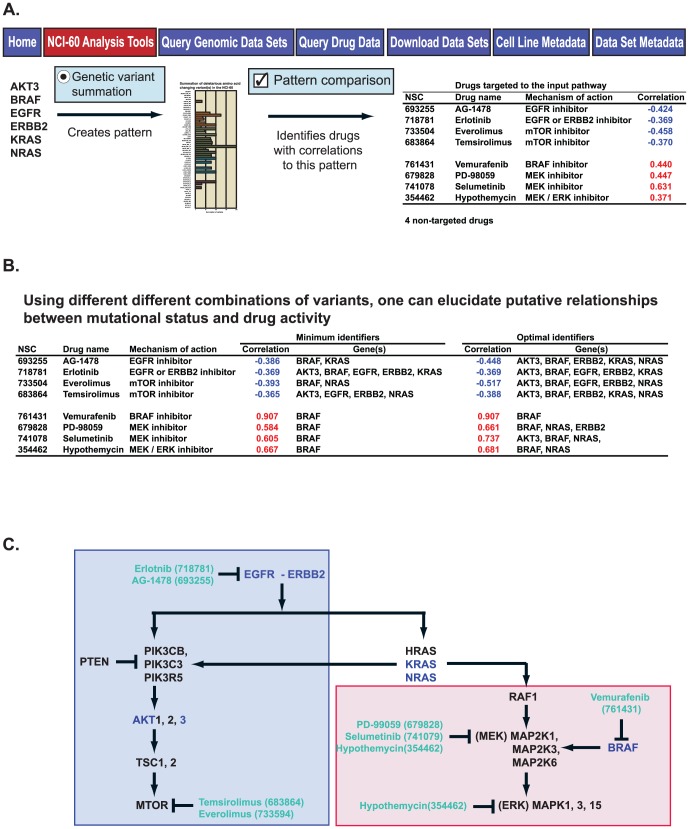
Use of the “Genetic variant summation” tool output for pharmacological exploration. **A**. The 6-gene input from Figure 6A yields a summation pattern for the NCI-60. Input of this pattern to the “Pattern comparison” tool [15] identifies 12 significantly correlated drugs with known mechanism-of-action, including 8 that target the input pathway. **B**. By using the different outputs from the “Genetic variant summation” tool from Figure 6D as inputs to “Pattern comparison”, one may identify the minimum and optimal identifiers for the 8 drugs that target the input pathway. **C**. The molecular pathway from which the input genes were selected, including the targets of the 8 correlated drugs from A and B. The red-filled and blue-filled boxes indicate the drugs that work better, or worse, respectively, in the presence of the genetic variants from A and B.

The “Totals” of the amino acid-changing, protein function-affecting variants as visualized in Figure 6D can be used as input for our previously described and publicly available “Pattern comparison” tool (also found under the NCI-60 Analysis Tools tab) to interrogate any given pathway for potential pharmacological impact [15]. An example is given for illustration in Figure 7. The pattern from the Figure 7A gene input list generated using the “Genetic variant summation” tool is input to the “Pattern comparison” tool. From this pattern, 12 drugs with known mechanisms of action showed significant correlations, eight of which target the pathway of the input genes. That is, drugs targeting that pathway are robustly enriched (p = 2.6×10^−6^) as accessed using the Fishers Exact 2-tailed test calculation [60]. By varying the input selection of genes, one may identify either the minimal or optimal grouping of genes whose genetic variants are correlated to these drugs activities (Figure 7B). Additionally, by viewing the drug gene interactions in the pathway context, one may determine those grouping of variants correlated to either increased (positive correlations, grouped within the red box in Figure 7C) or decreased (negative correlations, grouped within the blue box in Figure 7C) drug activities. BRAF activation is seen to have significant relationship to both sides of the pathway, although it is most robust for the BRAF, MEK, and ERK inhibitors (Figure 7C).

## Discussion

The present study provides novel insights into and tools for exploring both genetic variant status and pharmacological response, based on a systematic analysis of both. It also describes how the data can be readily accessed by non-bioinformatics users (Figure 1 and Figures 5–7). Figure 2, Table 1, and Table S1 variants (putatively protein function affecting and absent in the non-cancerous genomes) identify putatively cancer-associated functionally significant homozygous variants, and thus provide valuable models of cancer cell behavior and drug response in the presence of significant genetic alterations. This is informative in the context of both synthetic lethal studies, and the identification of putative gene knockout cell lines. Examples from Table 1 and Table S1 include a wide variety of cancer and pharmacology important genes, including drug targets (CKN2A, PTEN, MAP3K5), tumor suppressors (TP53, APC, VHL, CDH4), oncogenes (BRAF, KRAS, EGFR), and apoptosis (TRAF1 and PIK3R2), DNA damage response (FANCM, MLH1, TDP1), and drug efflux (ABCB1) genes. We have previously verified functional alteration for TDP1 [61].

The variant frequency ratios in the NCI-60 compared to non-cancerous genomes (Figure 3A) revealed inflection points at which variant frequencies markedly deviate from the non-cancerous genomes. Interestingly, the percentage of protein function affecting, amino acid changing variants (Figure 3B) increases as these ratios increase, implying functional relevance for the enriched subset. As these variants occur in the normal population, but at unusually high or low levels in individuals that have gone on to develop cancer, there is implication of cancer pre-disposing or protective functions. The presence of the 42 known cancer-driver genes presented in Table 2 strengthens this hypothesis.

The systematic consideration of pharmacological response led to the identification of the broad cellular diversity (in pharmacological response) visualized in Figure 4B. This implies the presence of some combination of causal molecular events; however their detailed identification is beyond the scope of the current study. Our variant versus compound assessment (Table 3 and Table S4A and B) identifies significant mathematical relationships between gene variants and compound activities. Both germline and somatic variants are included as our purpose is to reveal potential influences on pharmacological response regardless of this distinction.

**Table 3:** Variant combinitorials versus drug activity correlation^a^.

	Drug					Cells with variant(s)		Cells without variant(s)			
			Mechanism	Developmental		Have drug	No drug	Have drug	No drug		
Gene name	NSC ID^b^	Name	of action^c^	status	Response	response	response	response	response		MCC^d^
BRAF	761431	Vemurafenib	YK	FDA approved	Sensitivity	9	1	0		49	0.94
BRAF	354462	Hypothemycin	YK	Clinical trial	Sensitivity	9	2	6		43	0.62
BRIP1	127716	5-Aza-2′-deoxycytidine	DNMT	FDA approved	Sensitivity	3	0	4		40	0.62
DFFB	127716	5-Aza-2′-deoxycytidine	DNMT	FDA approved	Sensitivity	3	0	4		40	0.62
DNMT3B	118218	Fludarabine phoshate	Ds	FDA approved	Sensitivity	6	0	7		44	0.63
GTF3C4	36405	Chelerythrine	PKC,STK	Clinical trial	Sensitivity	3	1	1		44	0.73
HDAC10	679828	PD-98059	STK	Clinical trial	Sensitivity	6	0	8		45	0.60
HTRA2	409962	Carmustine	A7	FDA approved	Sensitivity	5	0	6		49	0.64
IDH1	719276	Fulvestrant	Ho	FDA approved	Sensitivity	3	1	2		52	0.64
IGF1R	127716	5-Aza-2′-deoxycytidine	DNMT	FDA approved	Sensitivity	3	0	4		40	0.62
IGF1R	36405	Chelerythrine	PKC,STK	Clinical trial	Sensitivity	3	0	1		45	0.86
JAK3	122758	Tretinoin	Ho	FDA approved	Sensitivity	3	0	1		55	0.86
JAK3	697979	Denileukin	IL	FDA approved	Sensitivity	3	0	2		55	0.76
MORF4L1	750690	Sunitinib malate	YK	FDA approved	Sensitivity	7	1	6		45	0.63
MUS81	105014	Cladribine	Ds	FDA approved	Sensitivity	9	1	7		42	0.64
MUS81	606869	Clofarabine	Ds	FDA approved	Sensitivity	8	0	9		42	0.62
RAD52	109724	Ifosfamide	A7	FDA approved	Sensitivity	5	1	4		41	0.63
SETD2	102816	Azacytidine	DNMT	FDA approved	Sensitivity	5	0	6		49	0.64
SLC12A8	366140	Pyrazoloacridine	Db	Clinical trial	Sensitivity	7	0	8		31	0.61
SLC17A9	365798	Fostamatinib disodium	YK	Clinical trial	Sensitivity	7	1	5		34	0.64
SLC22A16	105014	Cladribine	Ds	FDA approved	Sensitivity	10	2	6		41	0.64
SLC22A16	312887	Fludarabine phosphate	Ds	FDA approved	Sensitivity	9	3	5		43	0.61
SLC22A16	606869	Clofarabine	Ds	FDA approved	Sensitivity	10	2	7		40	0.61
STAT2	102816	Azacytidine	DNMT	FDA approved	Sensitivity	5	0	6		49	0.64
STAT2	34462	Uracil mustard	A7	FDA approved	Sensitivity	7	0	8		45	0.63
TET2	633782	Simvastatin	AM	FDA approved	Sensitivity	9	1	5		26	0.67
TNFSF11	127716	5-Aza-2′-deoxycytidine	DNMT	FDA approved	Sensitivity	3	0	4		40	0.62
UNC5B	376128	Dolastatin 10	Tu	Clinical trial	Sensitivity	8	2	5		35	0.62
ABCC10	141633	Homoharringtonine	Apo	FDA approved	Resistance	5	1	4		37	0.62
ABCC2	756645	Crizotinib	YK	Clinical trial	Resistance	8	0	9		42	0.62
CTNNA2	36405	Chelerythrine	PKC,STK	Clinical trial	Resistance	3	0	3		43	0.68
MCM2	608210	Vinorelbine tartrate	Tu	FDA approved	Resistance	3	0	4		50	0.63
POLD1	105014	Cladribine	Ds	FDA approved	Resistance	8	0	10		41	0.60
SERPINE2	38721	Mitotane	Ho	FDA approved	Resistance	3	1	0		39	0.86
SLC39A8	686288	Aminoflavone	Db	Clinical trial	Resistance	10	1	8		40	0.63
SLC4A1AP	752782	Pazopanib	YK	FDA approved	Resistance	3	0	4		46	0.63

a Input compounds (Figure 4A and Table S3) and amino acid changing variants compared by Matthew's correlation coefficient.

b National Service Center identifier.

c Drug mechanism of action abbreviations: A7 is alkylating at N-7 position of guanine; Apo is apoptotic, Db is DNA binder; DNMT is DNA methylttransferase inhibitor; Ds is DNA synthesis inhibitor; PKC is protein kinase C inhibitor; STK is serine threonine kinase; T1 is topoisomerase 1 inhibitor; T2 is topoisomerase 2 inhibitor; and YK is tyrosine kinase inhibitor.

d Matthew's correlation coefficient.

The Table S4A and B of significant relationships can be queried by either gene or drug, dependent on the user's interest, with specific pairings of interest easily visualized using the Genetic variant versus drug visualization tool (Figure 5). Examples from these tables verifying previously associated pairings include BRAF with i) vemurafenib (r = 0.939), ii) the MEK-ERK inhibitor hypothemycin (r = 0.621), and iii) the MEK inhibitor ARRAY-162 (NSCs 761431 354462, and 764042 respectively) [11], [13], [14], [62], [63], [64]. Prior literature also provides indirect prior evidence for multiple pairings. SETD2 is a histone methyl-transferase correlated (r = 0.637) to increased sensitivity in azacytidine (NSC102816). Closely related 5-aza-2′-deoxycytidine has been shown to mediate histone methylation levels [65]. IGF1R is an anti-apoptotic correlated (r = 0.627) to increased sensitivity for the TOP2 inhibitor XK-469 (NSC697887). The same gene has been previously associated with another TOP2 inhibitor, etoposide [66]. STAT2 is a pro-apoptotic correlated (r = 0.603) with increased sensitivity the DNA synthesis inhibitor aphidicolin glycinate (NSC303812). The same gene has been previously associated with another DNA synthesis inhibitor, 5-flourouracil [67]. RAD52 is a homologous recombination gene correlated (r = 0.629) with increased sensitivity for the A7 alkylating agent ifosfamide (NSC109724) (Figure 5C). Homologous recombination has been previously shown to be involved in the repair of A7 alkylating agents [68]. Our large-scale statistical assessment of the relationships between genes' variants and compounds' activities (Table 3 and Table S4A and B), present all results of the analysis, consistent with prior publications [13], [14].

Our web-based “Genetic variant versus drug visualization” tool (Figure 5) was designed to facilitate exploration of potential gene variants versus drug relationships. An example from the vemurafenib versus BRAF comparison (Figure 5C) is that one melanoma, LOXIMVI, although having the V600E alteration, has reduced sensitivity as compared to the other cell lines with that mutation. Thus LOXIMVI provides a test case for V600E containing melanomas that have resistance to vemurafenib, a frequent clinical problem. A second example comes from the ifosfamide versus RAD52 pairing (Figure 5C). It suggests that while the RAD52 Q221E and Y415X variants might contribute to increased drug sensitivity, the S346X does not (X is a stop codon).

The second pharmacogenomic CellMiner tool introduced in the present publication is the “Genetic Variant Summation” tool. Using the web-based “Genetic Variant Summation” tool shown in Figure 6A, one may quickly identify the summations for variants present for any gene, and for gene combinations of interest (examples presented in Figure 6D). Moreover, by using these summations as input into our “Pattern Comparison” tool [15], one in turn gains insight into relationships between variants and pharmacological response. In the presence of the genetic variants of the six genes from Figure 6A and 7A, the EGFR, ERBB2, and MTOR inhibitors (within the blue box in Figure 7C) work less well (have negative correlations), while the BRAF, MEK and ERK inhibitors (within the red box in Figure 7C) work better (have positive correlations). One may also gain insight into which genes are most influential, in this case BRAF for the BRAF, MEK and ERK inhibitors (Figure 7B, bottom half). Perhaps as importantly, one may identify mutations not significantly influential, such as those in KRAS and NRAS for the clinically important vemurafenib, and KRAS for selumetinib.

Taken in context with the CCLE and GDS databases, we believe the NCI-60 has both liabilities and assets. The limited number of cell lines renders difficult the detection and statistical assessment of possible effects of rare genetic alterations. Likewise, disease types absent within the set (for instance sarcomas and hepatocellular carcinomas) are not assessable. Offsetting this, is the unmatched array of clinically relevant drug and compound information available in the NCI-60 DTP database, which includes FDA-approved, clinical trial, known mechanism of action drugs, and a vast number of compounds, including natural products that can serve as seeds for novel anticancer classes [19]. The raw numbers for pharmacological comparison are: i) for CCLE, 24 drugs on less than 1000 cells yielding <24,000 responses, ii) for GDS, 138 drugs on less than 1000 cells yielding <138,000 responses, and iii) for the NCI-60 19,940 compounds for 60 cells yielding 1,196,400 responses. This combination of known and unstudied compounds is and will remain a unique asset for the field. Known drugs may be used to identify important novel molecular parameters in pharmacology, as was done for SLFN11 [69], [70]. The unstudied compounds are particularly useful when screening for any parameter for which drugs do not currently exist, as was done in the case of Ro5-3335 for core binding factor leukemia [71]. This aspect is entirely missing from both CCLE and GDS. An additional important consideration for these activity measurements are that the assays run for each group are different, with CCLE measuring ATP levels at 72–84 hours, GDS measuring either nucleic acid content or oxidation-reduction at 72 hours, and the DTP measuring total protein at 48 hours post treatment. As no one measurement type or time gives all information, these datasets should be considered to be complimentary, not exclusively informative. A second important asset for the NCI-60 is the broad scope and quality of the associated molecular data (see introduction), which will be difficult, or more accurately unfeasible to obtain for 1,000 cell line sets. These multiple forms of molecular data become invaluable for systems pharmacological approaches. Thus, the NCI-60 is and will remain an important resource in conjunction with the other cell line and upcoming patient tumor xenograft (PDX) databases.

The present document can be viewed as a compendium for using the NCI CCR CellMiner [23] and its associated NCI DTP [20] websites. One of our aims is to allow the broadest possible range of users to assess genomic variant status and compound activity in the NCI-60, and their relationships to one another in a systematic and intuitive fashion. The variant data provide a compilation of homozygous protein function affecting variants. It identifies variants that occur in the general population that are enriched or depleted in the context of cancer, implying a role in predisposition or protection with respect to cancer progression. The cell lines are revealed to have significant intrinsic variability for drug resistance, increasing their usefulness as pharmacological response models. All significant gene variant-drug correlations are identified, providing a unique resource for the field. An assessment of the total relative influences of genetic variants relative to their presence or absence in normal genomes is provided. Finally, the introduction of the “Genetic variant versus drug visualization” and “Genetic variant summation” web-based tools will enable the exploration of relationships between DNA variation and pharmacological response, both for single genes and groups of genes for non-informaticists. This will provide the opportunity to generate hypothesis, discover novel therapeutic agents, and gain insights based on cancer genomics.

## Supporting Information

Table S1 Homozygous, amino-acid changing, putative protein-function-affecting genetic variants present in the NCI-60, and absent in the 1000 Genomes and ESP5400. The full-length version of Table 1.
(XLSX)

Table S2 3,111 variants over or under-represented in the NCI-60 as compared to the ESP5400. The full-length version of Table 2.
(XLSX)

Table S3 19,940 Compounds included in the analysis.
(XLSX)

Table S4 **Table S4A and B.** Combinatorial analysis for amino acid changing variants that are significantly correlated to increased sensitivity (Table S4A) or resistance (Table S4B) to compounds. The full-length version of Table 3.
(XLSX)
